# Persistence of Immunity for Hepatitis B Virus among Heathcare Workers and Italian Medical Students 20 Years after Vaccination

**DOI:** 10.3390/ijerph16091515

**Published:** 2019-04-29

**Authors:** Luca Coppeta, Andrea Pompei, Ottavia Balbi, Ludovico M. De Zordo, Federica Mormone, Sara Policardo, Piergiorgio Lieto, Antonio Pietroiusti, Andrea Magrini

**Affiliations:** 1 Department of Occupational Medicine, University of Rome Tor Vergata, Viale Oxford 81, 00188 Roma, Italy; ottaventidue@hotmail.it (O.B.); dottordez@gmail.com (L.M.D.Z.); federica.mormone25@gmail.com (F.M.); sara.policardo@gmail.com (S.P.); piergiorgio.lieto@gmail.com (P.L.); pietroiu@uniroma2.it (A.P.); andrea.magrini@uniroma2.it (A.M.); 2 Department of Occupational Diseases, Toulouse University Hospital, Bâtiment Turiaf, Place du Dr Baylac, 31059, Toulouse Cedex 9, France; andpom15@libero.it

**Keywords:** HBV, healthcare workers, vaccination, booster, immunological memory

## Abstract

*Background*: Immunization of healthcare workers (HCWs) and medical students for the hepatitis B virus (HBV) is a crucial part of the hospital infection control programs. The aim of our study was to evaluate the persistence of anti-HBV specific antibodies in HCWs vaccinated during infancy or adolescence. *Methods*: Medical records of 734 consecutive subjects born after 1980 (481 females, 65.5% and 253 males, 34.5%) who underwent serological testing for anti-hepatitis B surface antibodies (anti-HBs) were evaluated. *Results*: A non-protective titer (<10 mUI) was found in 88/734 (12.0%) subjects; 84 (47.8%) of them received a booster dose of anti-hepatitis B vaccine and the anti-HBs titer of 58 subjects was measured 1 month after administration. A protective titer (anti-HBs >10 mIU/mL) was observed in almost 90% of subjects receiving the booster dose. *Conclusions*: A substantial percentage of HCWs had a non-protective anti-HBs titer at the time of the first employment, especially those vaccinated at birth age. However, the response to the booster dose showed that in these subjects, an anti-HBs titer <10 mIU/mL was due to the physiological decline of antibodies over the years. Therefore, primary immunization in childhood is highly effective and provides lasting immunity against HBV infection.

## 1. Background

Hepatitis B virus (HBV) infection is a major health concern and worldwide over 2 billion subjects (1/3 of the world population) have evidence of HBV infection [1,2,3].

HBV is a highly infectious virus, which is transmitted parenterally (needlestick, mucosal or non-intact skin exposure) [4] and remains infectious in the environment for at least seven days [5]. Percutaneous exposure (i.e., needlestick) is among the most efficient ways of transmission of HBV, however, this type of exposure represents a minority of HBV infections among healthcare workers (HCWs). In fact, the majority of HBV infections occur through childhood and perinatal transmission [6,7]. It has been estimated that 90% of anti-HBV vaccine coverage would be able to prevent 537,000–660,000 deaths each year worldwide [8,9].

HCWs are considered to be a population at high-risk to develop HBV infection due to the high transmissibility of the virus and the risk related to occupational injuries [10,11]. The World Health Organization (WHO) estimates that more than 300,000 HCWs are exposed every year to accidental percutaneous contact with contaminated fomites, and that about 66,000 of them become infected [10,11,12]. Thus, anti-HBV vaccination is recommended for all HCWs independently of job duty [13,14].

In recent statistics, HBV vaccination coverage among HCWs ranges between 46% and 74%, depending on their specific duty [13,15]; both of these values are substantially below the target of 90% anti-HBV vaccination coverage among HCWs, established by Healthy People 2020 program [13,16,17].

The HBV vaccine is considered effective, according to the reported data [18,19,20]: A vaccine-induced seroprotection was observed in approximately 95% of healthy children [21,22], about 92% of healthcare workers aged <40 years and about 84% of healthcare workers older than 40 years [23]. 

The anti-hepatitis B surface antibody (anti-HB) titer decreases over time according to age at vaccination: Approximately 16% of people vaccinated at the age of <1 year have detectable antibody levels ≥10 mIU/mL [24,25,26,27,28,29] at 18 years of vaccination, compared to 74% for those vaccinated at age ≥1 year [20,29,30,31,32,33,34,35,36,37].

Available evidence suggests that protection against hepatitis B infection in immunocompetent responders persists for ≥22 years [18,19,38].

The HBV vaccination in Italy became compulsory after June 1991 for two cohorts of children (at birth and at 12 years old). After a three-dose vaccination, more than 95% of subjects develop a protective anti-HBs titer, while 5% of vaccinated remain non-responders. Despite the anti-HBs titer decreasing over time, which can reach values <10 mIU/mL 5–20 years after vaccination, these subjects seem to be protected against HBV infection due to the presence of immunological memory [39,40].

The current Italian National Vaccine Prevention Plan (PNPV), strongly recommends anti-HBV vaccination for all HCWs and medical students, before starting the activities at risk in all health facilities [41]. Three doses of anti-HBV vaccine (at time 0, 1 and 6–12 months) should be offered to previously unvaccinated, HCWs. A four-dose schedule (at month 0, 1, 2, and 12) must be administered in case of a documented exposure to potentially infectious material. The seroconversion should be verified after the third or the fourth dose to confirm protection [42,43,44,45]. Anti-HBs serological screening is recommended for all subjects born after 1980, although presumably, they are vaccinated against HBV in the case of being engaged in activities at risk. An administration of the booster dose is strongly recommended for HCWs with anti-HBs <10 mIU/mL if lacking certification of protective antibody titers, and anti-HBs dosage one month after booster administration should be performed [42]. Medical students are included in the current recommendation.

## 2. Methods

The aim of our study was to evaluate serological immunity against HBV in HCWs and medical students born after 1980, in order to verify the persistence of protective anti-HBs over time. 

In this retrospective study, we evaluated medical records of 734 HCWs and medical students who underwent medical examination before attending the internship at the Tor Vergata University Hospital in Rome (PTV) between January and December 2018.

All subjects were evaluated for anti-HBs. Those who had anti-HB concentrations lower than 10 mIU/mL were offered a booster dose of recombinant anti-hepatitis B vaccine (available on the market) and the anti-HB titer was checked again one month later.

Subjects positive for hepatitis B (HB) antigen (Ag) or anti-hepatitis B core antigen antibody (anti-HBc) have been excluded from the study.

For each subject, we recorded the following data: The sex, date of birth and levels of anti-HBs if performed at any time after vaccination.

The subjects were thereafter categorized into two groups:

- People having anti-HB levels higher than 10 mIU/mL (generally considered as protective concentrations)
- People having anti-HB levels lower than 10 mIU/mL.

Subjects in the latter group were offered counselling on vaccination and those accepting additional vaccination were given a booster dose of monovalent HBV vaccine.

The serological tests were carried out with ECLIA (electrochemiluminescence immunoassay) by the microbiology laboratory of our university.

For the purpose of this study, access to clinical data was restricted to the researchers participating in the study. Afterward, all personal data was removed from the analytical database. 

All procedures performed in this study were approved by the Ethical Committee of our institution and the informed consent was obtained by people included in the study. 

Analyses were performed using STATA^®^ software (Version number 11). The data were expressed as the mean ± standard deviation (SD). Assessments of statistical significance between mean titers were conducted using the Mann–Whitney U test for continuous variables with a non-normal distribution. Each independent variable (i.e., gender, time from vaccination, age at vaccination) was first tested for univariate association with the dependent variable using Fisher’s exact test for dichotomous variables. Variables with a *p*-value <0.05 in the univariate analysis were entered into multivariate logistic regression models, using a backward elimination method, to explore the relative contributions of the various characteristics. Results were considered statistically significant at a *p*-value <0.05.

## 3. Results

A total of 734 individuals were involved, comprising 481 females (65.5%) and 253 males (34.5%); the main characteristics of the subjects are shown in Table 1.

The median time elapsed between vaccination and our study was 20.5 years (±3.69). No participants had written documentation of a previous anti-HB titer evaluation. Therefore, we checked the antibody concentration during the pre-employment screening for all the subjects enrolled in the study.

We set the antibody concentration threshold of 10 mIU/ml to divide HCWs and students’ baseline serological tests into two groups:

- Group 1: 646/734 (88.0%) subjects, with antibody levels higher than 10 mIU/mL. This group was, therefore, considered to be immunized against HBV
- Group 2: 88/734 (12.0%) subjects, with antibody level slower than 10 mIU/mL. This group was, therefore, considered to be at risk of HBV infection, in the case of exposure.

We found significant differences between the average titer in subjects vaccinated during childhood and those vaccinated at 12 years, and between male and female subjects (*p* < 0.05), as shown in Table 2.

These differences were statistically significant also in multivariate analysis, controlling for time elapsed from the vaccination (more or less than 20 years) as a possible confounder.

**Figure 1:**
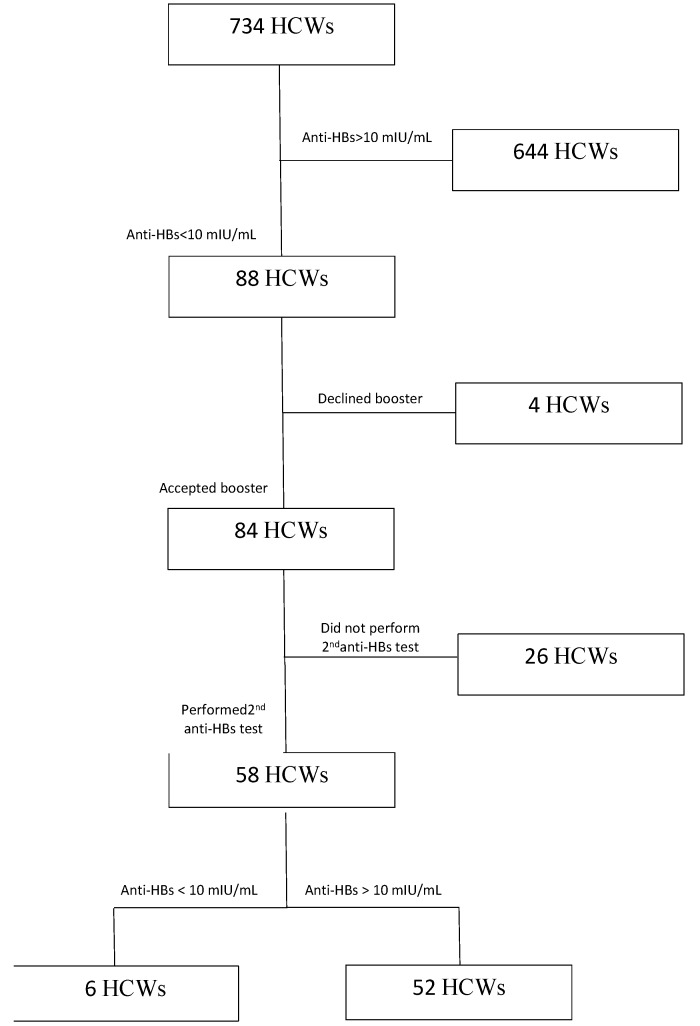
Study participants’ flow chart.

In Group 2, 84 of 88 subjects (95.5%) accepted the administration of the booster dose. These subjects were checked for antibody response 4–6 weeks later, according to the current guidelines [44]. A total of 26 subjects dropped out, therefore, the antibody response was performed in 56 subjects (69% of the sample). Successful immunization (titer higher than 10 mIU/mL) was observed in 52/58 (89.7%) (see the related flowchart in Figure 1). 

All non-responders belonged to the group of people vaccinated at adolescence. 

## 4. Discussion

Protection against infections remains a priority for HCWs. The need to keep HCWs immune to infectious diseases is a significant objective of the National Healthcare Service, pursued through a prevention and control plan. In order to preserve the well-being of healthcare professionals and, consequently, of patients interacting with them, the correct use of protection measures, such as immunizing agents, is fundamental.

Our study shows suboptimal levels of protection among HCWs vaccinated during infancy or adolescence. The prevalence of a protective anti-HB titer in pre-employment screening was statistically associated with gender and the age of vaccination. Subjects vaccinated at an age of one year were significantly less protected than HCWs vaccinated at 12 years, even after controlling for the possible confounding effect of time elapsed from the vaccination. 

Although HBV vaccination has been carried out for several years, the debate on the duration of protection is still open. Furthermore, the fact that the post-immunization (four weeks after the first series of vaccinations) is not always available and the questions on the real need and effectiveness of booster doses still remain unanswered. Experts have been dealing with these issues since the creation of the universal HBV vaccine policy for infants, children and adolescents. In 1996, notably five years after the institution of mandatory vaccination of infants and 12-year-old children, research was conducted to verify the persistence of anti-HB concentrations >10 mIU/mL in the population who underwent the vaccine. It was found that 92.9% of children and 94.1% of teenagers were protected against HBV (anti-HB titer >10 mIU/mL). In adolescents, the antibody levels were much higher than in children [46].

Another investigation regarded children and recruits of the Italian Air Force that underwent vaccination more than a decade before; the results showed that anti-HB concentrations were protective in 64% of kids and 89% of recruits [47].

Iranian research checked the anti-HB titers of 300 adults, two decades after the first compulsory vaccination. Here, only 37% of the subjects had protective antibody levels (>10 mIU/mL). The rate of protection increased to 97.1% after the administration of a booster dose. These results are consistent with the finding of our study—regarding response of 89.7% to the booster dose—and confirm the persistence of long-term immunological memory in vaccinated individuals with low levels of anti-HBs [48].

In fact, in our study, most of the unprotected subjects become protected after receiving a booster dose of HBV vaccine. It is interesting to note the six subjects with lack of immunization after the booster dose had received a vaccination at the age of 12 years, a finding consistent with a more rapid decline of anti-HB titer among people vaccinated in infancy, as previously reported [49].

Although in the Italian Ministry of Health recommendations there are currently no indications to test the antibody titer after administration of the complete HBV vaccination cycle in the general population, the results of our study highlight that up to 20% of people tested 20 years after the primary vaccination had a titer <10 mIU/mL, showing a potential lack of protection, at an age in which the exposure to HBV from non-professional sources may happen (sexual activity, drugs abuse, etc.).

Furthermore, our study shows a gender-based HBV titer difference. A sex-difference in antibody response—in which female was greater than male—was previously reported in the literature [50]. As immunity has been observed to be sexually dysmorphic [51] in both animals and humans, it might be expected that sex differences in immune responses would be observed also with the HBV vaccine in humans.

This research has some limitations: It was a retrospective, observational study, and we had no data available on the formulation and dosage of the primary series vaccination. Moreover, not all of the seronegative HCWs returned to receive the booster dose and this fact could have influenced the outcomes. Since we did a cross-sectional study, we cannot validate the hypothesis that the decrease in subjects vaccinated in infancy is faster than that of those vaccinated in adolescence, because we could not test the trend of antibody titer in the same subject over time.

## 5. Conclusions

This investigation offers additional knowledge and reflections on the persistence, in people, of anti-HBV immunity approximately two decades after vaccination from early childhood. The outcomes of our study shows a substantial percentage of HCWs having a non-protective anti-HBs titer at the time of first employment (mostly in those vaccinated at birth age). Nevertheless, the response to a booster dose in those subjects demonstrates that it is due the physiological decline of antibodies over years and that the primary immunization at childhood is highly effective and provides a long-lasting immunity against HBV infection. In our study, according to data from the literature, vaccine responder HCWs do not need a retesting for at least twenty years after evidence of a protective anti-HB titer at the post-vaccination test.

## Figures and Tables

**Table 1:** Demographic characteristics of healthcare workers (*n* = 734).

Characteristics	*N* (%)	Mean Age (± SD)	Mean Titer (± SD)
Total number	734 (100)	29.88 ± 4.00	353.47 ± 390.55
Gender			
Male	253 (34.5)	29.35 ± 4.01	317.49 ± 377.31
Female	481 (65.5)	30.16 ± 3.97	372.29 ± 396.38
Time from vaccination			
<20 years	376 (51.2)	28.42 ± 1.85	359.05 ± 392.25
≥20 years	358 (48.8)	31.41 ± 4.97	346.23 ± 388.82
Age of vaccination			
1 year old	155 (21.1)	24.68 ± 1.88	232.77 ± 341.69
12 years old	579 (78.9)	31.27 ± 3.19	375.78 ± 395.15

**Table 2:** Association between gender, age of birth, time from vaccination and protection (titer > 10 UI/mL). Univariate and multivariate analysis.

Variables	Total	Titer >10		*p*-Value		
		Percent	Number	Percent	Univariate	Multivariate
Gender						
Female	481	67.3	435	90.3	<0.05	<0.05
Male	253	32.7	211	83.4		
Age of vaccination						
1 year old	114	13,.6	88	77.2	<0.05	<0.05
12 years old	620	86.4	558	90.0		
Time from vaccination						
<20 years	416	55.7	360	86.5	=0.09	
≥20 years	318	44.3	286	89.9		

## Abbreviations

HBV: Hepatitis B Virus; WHO: World Health Organization; HCW: Heathcare Workers; PNPV: Piano Nazionale di Prevenzione Vaccinale; PTV: Tor Vergata University Hospital; ECLIA: Electro ChemiLuminescence Immuno Assay.

## Declarations

Ethics approval and consent to participate: All procedures performed in this study were approved by the ethical committee of Policlinic Tor Vergata. Consent for publication: Written consent was obtained from all participants. Availability of data and material: The datasets used and/or analyzed during the current study are available from the corresponding author on reasonable request.
